# Cabergoline monotherapy in polycystic ovary syndrome patients with elevated prolactin: a viable option?

**DOI:** 10.1007/s12020-025-04279-8

**Published:** 2025-05-21

**Authors:** Aslı Sıgınır, Hayri Bostan, Emre Sedar Saygılı, Ceren Tufan, Ersen Karakılıc

**Affiliations:** 1 Department of Internal Medicine, Canakkale Onsekiz Mart University Faculty of Medicine, Canakkale, Türkiye; 2 Department of Endocrinology and Metabolism, Canakkale Mehmet Akif Ersoy State Hospital, Canakkale, Türkiye; 3 Department of Internal Medicine, Division of Endocrinology and Metabolism, Canakkale Onsekiz Mart University Faculty of Medicine, Canakkale, Türkiye

**Keywords:** Cabergoline, Polycystic ovary syndrome, Prolactinoma, Androgens, Menstrual irregularities, Hirsutism

## Abstract

**Purpose:**

Cabergoline is widely used to treat hyperprolactinemia, but its effects on polycystic ovary syndrome (PCOS) remain unclear. Since hyperprolactinemia is present in nearly 30% of PCOS cases, this study aims to assess the impact of cabergoline on androgen levels and clinical outcomes in PCOS with elevated prolactin cases, discussing these findings with the results in prolactinoma cases.

**Methods:**

A total of 66 women aged 18–40 were included in this retrospective cohort study, with 36 in the PCOS with elevated prolactin group (median 24.0 (22.0–27.5) years) and 30 in the prolactinoma group (median 28.0 (23.7–33.0) years). Only patients who had been started on cabergoline treatment and had available follow-up data were included. Hormonal profiles and clinical findings, including hirsutism, and menstrual cycle regularity, were assessed before and after cabergoline treatment.

**Results:**

After cabergoline treatment, significant reductions in prolactin and total testosterone levels were observed in both groups. In the PCOS group, total testosterone decreased from 0.65–0.49 ng/mL (*p* < 0.001) and dehydroepiandrosterone-sulphate levels from 407.5–301.0 µg/dL (*p* < 0.001). In the prolactinoma group, total testosterone decreased from 0.39–0.29 ng/mL (*p* < 0.001). Menstrual irregularities improved markedly in both groups, with prevalence decreasing from 83.3–5.6% in PCOS group and from 80.0–10.0% in the prolactinoma group (*p* < 0.001). Furthermore, in PCOS group, the prevalence of hirsutism was decreased from 86.1–61.1% (*p* = 0.007).

**Conclusion:**

Cabergoline is effective in lowering prolactin and androgen levels while improving menstrual regularity in both PCOS and prolactinoma patients, highlighting its potential as a valuable therapeutic option for patients with PCOS with elevated prolactin.

## Introduction

Polycystic ovary syndrome (PCOS) is a prevalent disorder among women of reproductive age, primarily marked by chronic anovulation and hyperandrogenemia. Despite not being a rare disease, the exact pathophysiology of PCOS remains unclear [1]. PCOS is acknowledged as a multisystemic disorder influenced by a combination of genetic predisposition and environmental factors. Abnormalities in gonadotropic and steroidogenic hormones have been implicated in its development [2].

The ongoing uncertainty regarding the pathophysiology and diagnosis of PCOS also extends to its treatment, as no standardized approach has been universally established. Management must be individualized, with treatment goals aimed at addressing hyperandrogenism, preventing endometrial hyperplasia, managing metabolic risks, and improving fertility in women desiring pregnancy [3, 4]. First-line treatment often includes lifestyle modifications, particularly for overweight patients [5]. While combined oral contraceptives (COCs) are commonly prescribed to regulate menstrual cycles and alleviate hyperandrogenic symptoms, their use is limited by side effects, including an increased risk of venous thromboembolism, particularly in patients with obesity or a family history of thromboembolism [6, 7]. Additionally, COCs are not suitable for women seeking fertility. Other treatment options, including anti-androgenic agents such as spironolactone, cyproterone acetate, and finasteride, are limited by their potential side effects. Spironolactone is most commonly associated with hyperkalemia, which can restrict its use. Hepatotoxicity and teratogenic risks are also other shortcomings of these medications, making them unsuitable for women who wish to conceive [7]. Metformin has shown some benefit, particularly in patients with insulin resistance, but its overall efficacy remains a subject of debate [8]. These limitations highlight the need for alternative therapeutic options.

Studies involving patients diagnosed with PCOS based on the Rotterdam criteria have reported a hyperprolactinemia prevalence ranging from 11.6–37% [9, 10]. While prolactin (PRL) levels in PCOS are generally lower than those observed in prolactinoma cases, a differential diagnosis is occasionally required to rule out prolactinoma [9]. On the other hand, hyperandrogenemia is not commonly associated with female prolactinoma cases. Nevertheless, both conditions often lead to menstrual irregularities and hirsutism.

Cabergoline (CAB), a dopamine agonist (DA), is the first-line treatment for prolactinoma and has been used for many years with a well-established safety profile [11]. CAB restores menstrual cycles in 97% of female prolactinoma patients [12]. Given this success, CAB therapy may also provide clinical improvements for PCOS patients with moderate PRL elevation. While a limited number of studies have shown positive outcomes in this context [13, 14], the use of CAB in hyperprolactinemic PCOS patients is not yet a guideline-recommended approach. In addition, most research on the effects of CAB on androgen levels has focused on male prolactinoma patients, resulting in limited knowledge about its influence in female populations. Therefore, the current study aimed to evaluate the laboratory and clinical outcomes of hyperprolactinemic PCOS and microprolactinoma patients treated with CAB.

## Materials and methods

### Study population and design

This retrospective cohort study was conducted at the Endocrinology Unit of Çanakkale Onsekiz Mart University, Faculty of Medicine. Patients of reproductive age diagnosed with PCOS with elevated PRL or prolactinoma and treated with CAB were reviewed in our clinic between March 2018 and March 2024. Although a defined indication for the use of DAs in patients with PCOS has not yet been established, the U.S. Food and Drug Administration (FDA) has approved the use of CAB for idiopathic hyperprolactinemia [15]. Based on the assumption that hyperprolactinemia in patients with PCOS may be independent of the disease itself and that previous studies on this subject encourage the use of DA [13, 14], CAB therapy has been administered in our clinic, particularly in patients with coexisting elevated PRL and androgen levels for a short duration, typically up to six months. If menstrual cycles were restored during this period, a trial discontinuation of the treatment was undertaken. However, if hyperandrogenism and menstrual irregularities persisted despite normalized PRL levels, we transitioned to standard therapies, such as COCs and/or antiandrogens.

A total of 66 women aged 18–40 with hyperprolactinemia were included in this study. The PCOS group consisted of 36 women patients who had initiated CAB treatment due to hyperprolactinemia. The prolactinoma group included 30 women patients with microprolactinoma who had also started CAB treatment during the same period. Due to the retrospective design, pre- and post-treatment assessments were based on the first visit prior to treatment initiation and the last documented follow-up during CAB therapy. Only patients who received regular CAB treatment were included in the analysis. Exclusion criteria included postmenopausal women, those diagnosed with cancer, severe comorbidities, autoimmune disorders, or those who were pregnant or breastfeeding. Participants who had used medications affecting PRL or androgen levels—such as antidepressants, antipsychotics, oral contraceptives, antiandrogens, glucocorticoids, or metformin—within the last six months were also excluded.

### Data collection

Demographic data, laboratory results, medical history, and medication use were obtained from the hospital information system. Clinical information, including the duration of CAB treatment, the presence of menstrual irregularities, galactorrhea, acne, and hirsutism, was also collected. All data were recorded in pre-designed study forms, assigned unique identification numbers, and archived. The collected information was later digitized for statistical analysis.

### Diagnostic criteria

The diagnosis of PCOS was made according to the 2003 Rotterdam criteria, which require the presence of at least two of the following: oligo-anovulation, clinical and/or biochemical hyperandrogenism, and polycystic ovarian morphology [16]. PRL levels were measured twice on separate days at 09:00 AM to confirm the presence of hyperprolactinemia. If the values were slightly above the upper limit of the reference range, a cannulated PRL test was performed to exclude stress-induced elevations. Afterward, PCOS patients with elevated PRL levels underwent magnetic resonance imaging (MRI) screening of the pituitary gland to rule out prolactinoma. Additionally, thyroid function tests, beta-HCG measurements, and a polyethylene glycol (PEG) precipitation test to exclude macroprolactinemia were conducted. A diagnosis of PCOS with elevated PRL was made only after the completion of these thorough assessments.

For hyperprolactinemic patients with pituitary adenomas identified on MRI, after excluding other potential causes (e.g., pregnancy, hypothyroidism, medication use, or macroprolactinemia), the diagnosis of prolactinoma was made following the current guidelines [17].

Amenorrhea was defined as the absence of menstrual bleeding for more than three months in non-pregnant individuals, and oligomenorrhea was defined as fewer than nine menstrual cycles per year. Recovery from menstrual irregularity following CAB treatment was determined by the resumption of menstrual cycles in patients with amenorrhea. Clinical hirsutism was confirmed in patients with a Ferriman-Gallwey (FG) score of ≥8.

### Laboratory assessments

Hormonal measurements, including dehydroepiandrosterone-sulphate (DHEA-S) (normal range (NR): 65.1–368 µg/dL), total testosterone (NR: 0.08–0.48 ng/mL), thyroid-stimulating hormone (TSH) (NR: 0.27–4.2 µIU/mL), PRL (NR: 4.79–23.3 ng/mL), insulin (NR: 2.6–24.9 µIU/mL), estradiol, follicule-stimulating hormone (FSH), and luteinizing hormone (LH) were analyzed using the Roche Cobas E601 Immunology Analyzer (Roche Diagnostics GmbH, Mannheim, Germany) via the electrochemiluminescence immunoassay method. Glucose levels were measured using the hexokinase method on the C501 analyzer. Insulin resistance was calculated using the HOMA-IR (Homeostatic Model Assessment of Insulin Resistance) formula: [(fasting serum insulin (µIU/mL) x fasting plasma glucose (mg/dL)) / 405].

### Data analysis

Statistical analyses were performed using the SPSS software package, version 20.0. The normality of continuous variables was evaluated using the Kolmogorov-Smirnov test. Since all continuous variables show a non-normal distribution, they are presented as medians with 25 and 75th percentiles (interquartile ranges), and the Mann-Whitney U test was employed to compare these variables between groups. Categorical variables are presented as percentages (%) and frequencies (n), and the Chi-square test was used to evaluate associations between categorical variables. In cases where the number of variables was less than five in any group, Fisher’s Exact test was applied. For within-group analyses of pre- and post-treatment continuous variables, the Wilcoxon test was used for non-normally distributed data, while the Paired Samples T-test was employed for normally distributed data. The McNemar test was used to analyze dependent categorical variables, and the Exact McNemar test was applied when fewer than five variables were present in the cells. A significance level of *p* < 0.05 was considered statistically significant for all tests.

## Results

### Comparisons of baseline characteristics across the groups

A total of 66 participants were included in the study, with 36 in the PCOS with elevated PRL group and 30 in the prolactinoma group. The median age was significantly lower in the PCOS group compared to the prolactinoma group (24.0 (22.0–27.5) vs. 28.0 (23.7–33.0) years, respectively; *p* = 0.013). In terms of hormonal levels, total testosterone was significantly higher in the PCOS group compared to the prolactinoma group (0.65 (0.54–0.85) vs. 0.39 (0.22–0.5) ng/mL, respectively; *p* < 0.001). However, there were no statistically significant differences between the two groups in DHEA-S, estradiol, FSH, LH, TSH, insulin, glucose, or HOMA-IR levels. As expected, PRL levels were significantly elevated in the prolactinoma group compared to the PCOS group (57.0 (39.0–99.5) vs. 40.0 (28.2–61.5) ng/mL, respectively; *p* = 0.003). Clinically, hirsutism and acne were significantly more common in the PCOS with elevated PRL group than in the prolactinoma group, with 86.1% of PCOS patients exhibiting hirsutism (*p* < 0.001) and 22.2% reporting acne (*p* = 0.006). The duration of cabergoline use did not differ significantly between the groups, and the majority of patients in both groups used a weekly dose of 0.5 mg (*p* = 0.58) (Table 1).

**Table 1:** Distribution of demographic, clinical, and laboratory parameters in prolactinoma and PCOS groups

*Parameter*	Prolactinoma group *(n* = *30)*	PCOS group *(n* = *36)*	*P*-value^*^
Age (years), median (IQR)	28.0 (23.7–33.0)	24.0 (22.0–27.5)	**0.013**
DHEA-S (ug/dL), median (IQR)	227.0 (206.0–400.5)	407.5 (247.5–534.2)	0.16
Total testosteron (ng/mL), median (IQR)	0.39 (0.22–0.5)	0.65 (0.54–0.85)	**<0.001**
Estradiol (pg/mL), median (IQR)	61.0 (37.5–135.5)	55.0 (45.0–90.0)	0.99
FSH (mIU/mL), median (IQR)	6.1 (3.8–8.4)	6.3 (5.5–7.4)	0.97
LH (mIU/mL), median (IQR)	8.7 (5.6–10.8)	10.6 (7.2–16.0)	**0.043**
TSH (uIU/mL), median (IQR)	2.7 (1.5–3.1)	2.6 (1.9–3.1)	0.56
Prolactin (ng/mL), median (IQR)	57.0 (39.0–99.5)	40.0 (28.2–61.5)	**0.003**
Insulin (uIU/mL), median (IQR)	14.0 (4.8–19.4)	10.7 (8.1–14.0)	0.36
Glucose (mg/dL), median (IQR)	94.0 (91.0–96.0)	87.0 (82.7–93.0)	0.32
HOMA-IR, median (IQR)	3.25 (1.08–4.74)	2.49 (1.67–3.01)	0.30
Hirsutism, n (%)	5 (16.7)	31 (86.1)	**<0.001**
Acne, n (%)	0 (0.0)	8 (22.2)	**0.006**
Menstrual irregularity, n (%)	24 (80.0)	30 (83.3)	0.73
Elevated total testosterone, n (%)	8(26.7)	33(91.7)	**<0.001**
Elevated DHEA-S, n (%)	4(30.8)	17(53.1)	0.205
Duration of cabergoline use (months), median (IQR)	4.0 (2.0–12.2)	3.0 (2.0–5.0)	0.13
Weekly dose of cabergoline (mg), n (%)			
0.5 mg/week, n (%)	23 (76.7)	29 (80.5)	0.58
1 mg/week n (%)	7 (23.3)	7 (19.5)	

*DHEA-S* dehydroepiandrosterone-sulphate, *HOMA-IR* homeostatic model assessment of insulin resistance, *FSH* follicule-stimulating hormone, *LH* luteinizing hormone, *PCOS* polycystic ovary syndrome, *TSH* thyroid-stimulating hormone

^*^Continuous variables were analyzed using the Mann–Whitney U test. Categorical variables were compared using the χ² test. Fisher’s Exact test was used when the number of variables in any group was less than five. Statistically significant results are indicated in bold

### Before and after treatment in PCOS with elevated prolactin

**Fig. 1:**
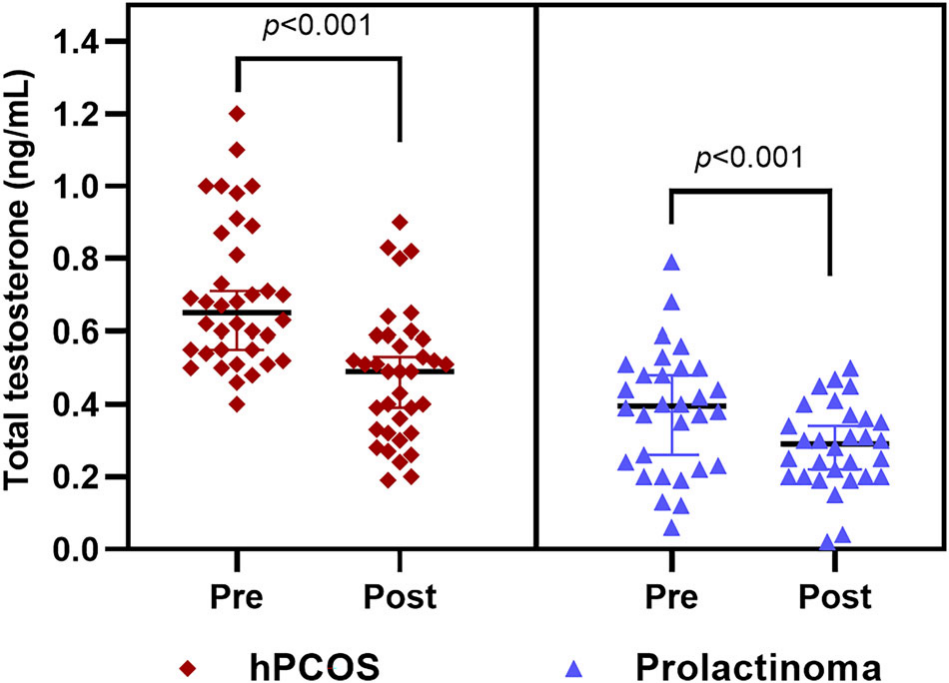
Comparison of pre- and post-treatment total testosterone levels in hypeprolactinemic PCOS and prolactinoma groups. The scatter plot represents median total testosterone levels and 95% confidence interval for both groups before and after treatment. A significant reduction in total testosterone levels was observed in both the PCOS group (*p* < 0.001) and the prolactinoma group (*p* < 0.001) following treatment. hPCOS: Policyctic ovary syndrome with elevated prolactin

In the PCOS group, significant reductions were observed in DHEA-S (from 407.5 (247.5–534.2) to 301.0 (210.7–405.7) µg/dL, *p* < 0.001), total testosterone (from 0.65 (0.54–0.85) to 0.49 (0.32–0.58) ng/mL, *p* < 0.001) (Fig. 1), and PRL levels (from 40.0 (28.2–61.5) to 1.1 (0.4–11.2) ng/mL, *p* < 0.001) following treatment. In addition, 4 patients of PCOS showed a decline in PRL without a corresponding decrease in DHEA-S, and 1 patient demonstrated no reduction in total testosterone despite a decline in PRL levels. Other parameters, including estradiol, FSH, LH, TSH, insulin, glucose, and HOMA-IR, showed no statistically significant changes post-treatment. Clinically, significant improvements were observed in menstrual irregularities, with their prevalence decreasing from 83.3–5.6% (*p* < 0.001), and in hirsutism, with prevalence declining from 86.1–61.1% (*p* = 0.007) (Table 2). The median follow-up duration for the 9 patients whose FG scores were <8 after CAB treatment was 4.5 months (3.0–7.5), whereas the follow-up duration for patients whose hirsutism did not improve was 3.0 months (1.0–4.0) (*p* = 0.037).

**Table 2:** Comparison of clinical and laboratory parameters in PCOS cases before and after cabergoline treatment

*Parameter*	Before treatment (*n* = 36)	After treatment (*n* = 36)	*P*-value^*^
DHEA-S (ug/dL), median (IQR)	407.5 (247.5–534.2)	301.0 (210.7–405.7)	**<0.001**
Total Testosteron (ng/mL), median (IQR)	0.65 (0.54–0.85)	0.49 (0.32–0.58)	**<0.001**
Estradiol (pg/mL), median (IQR)	55.0 (45.0–90.0)	67.5 (45.7–113.2)	0.15
FSH (mIU/mL), median (IQR)	6.3 (5.5–7.4)	5.3 (4.1–6.5)	0.06
LH (mIU/mL), median (IQR)	10.6 (7.2–16.0)	10.6 (7.2–18.0)	0.66
TSH (uIU/mL), median (IQR)	2.6 (1.9–3.1)	2.1 (1.2–2.8)	0.05
Prolactin (ng/mL), median (IQR)	40.0 (28.2–61.5)	1.1 (0.4–11.2)	**<0.001**
Insulin (uIU/mL), median (IQR)	10.7 (8.1–14.0)	11.5 (8.3–12.8)	0.60
Glucose (mg/dL), median (IQR)	87.0 (82.7–93.0)	89.0 (84.0–93.5)	0.39
HOMA-IR, median (IQR)	2.49 (1.67–3.01)	2.49 (1.48–2.83)	0.90
Hirsutism, *n* (%)	31 (86.1)	22 (61.1)	**0.007**
Menstrual irregularity, *n* (%)	30 (83.3)	2 (5.6)	<**0.001**
Acne, *n* (%)	8 (22.2)	7 (19.4)	0.56

*DHEA-S* dehydroepiandrosterone-sulphate, *HOMA-IR* homeostatic model assessment of insulin resistance, *FSH* follicule-stimulating hormone, *LH* luteinizing hormone, *PCOS* polycystic ovary syndrome, *TSH* thyroid-stimulating hormone

^*^The Wilcoxon signed-rank test was used for non-normally distributed data. Dependent categorical variables were analyzed using the McNemar test, and the Exact McNemar test was employed when cell counts were less than five. Statistically significant results are indicated in bold

### Before and after treatment in prolactinoma

In the prolactinoma group, significant reductions were observed in total testosterone levels (from 0.39 (0.22–0.5) to 0.29 (0.20–0.36) ng/mL, *p* < 0.001) (Fig. 1) and PRL levels (from 57.0 (39.0–99.5) to 10.0 (2.0–17.5) ng/mL, *p* < 0.001) after cabergoline treatment. Other hormonal markers, including DHEA-S, estradiol, FSH, LH, TSH, insulin, glucose, and HOMA-IR, showed no significant changes post-treatment. Clinically, the prevalence of menstrual irregularities decreased significantly from 80–10% (*p* < 0.001), while the rate of hirsutism remained relatively stable (from 16.7–10%, *p* = 0.50) (Table 3).

**Table 3:** Comparison of clinical and laboratory parameters in prolactinoma cases before and after cabergoline treatment

*Parameter*	Before treatment (*n* = 30)	After treatment (*n* = 30)	*P*-value^*^
DHEA-S (ug/dL), median (IQR)	227.0 (206.0–400.5)	249.5 (136.5–344.2)	0.19
Total Testosteron (ng/mL), median (IQR)	0.39 (0.22–0.50)	0.29 (0.20–0.36)	**<0.001**
Estradiol (pg/mL), median (IQR)	61.0 (37.5–135.5)	93.5 (62.0–144.2)	0.12
FSH (mIU/mL), median (IQR)	6.1 (3.8–8.4)	5.1 (4.1–8.7)	0.65
LH (mIU/mL), median (IQR)	8.7 (5.6–10.8)	9.6 (6.7–14.6)	0.07
TSH (uIU/mL), median (IQR)	2.7 (1.47–3.1)	2.6 (1.67–3.2)	0.45
Prolactin (ng/mL), median (IQR)	57.0 (39.0–99.5)	10.0 (2.0–17.5)	**<0.001**
Insulin (uIU/mL), median(IQR)	14.0 (4.8–19.4)	10.3 (8.0–20.7)	0.18
Glucose (mg/dL), median (IQR)	94.0 (91.0–96.0)	93.0 (86.2–101.0)	0.18
HOMA-IR, median (IQR)	3.20 (1.08–4.74)	2.30 (1.65–4.76)	0.18
Hirsutism, n (%)	5 (16.7)	3 (10.0)	0.16
Menstrual irregularity, n (%)	24 (80.0)	3 (10.0)	**<0.001**

*DHEA-S* dehydroepiandrosterone-sulphate, *HOMA-IR* homeostatic model assessment of insulin resistance, *FSH* follicule-stimulating hormone, *LH* luteinizing hormone, *TSH* thyroid-stimulating hormone

^*^The Wilcoxon signed-rank test was used for non-normally distributed data. Dependent categorical variables were analyzed using the McNemar test, and the Exact McNemar test was employed when cell counts were less than five. Statistically significant results are indicated in bold

## Discussion

The present study demonstrated that CAB effectively reduced androgen levels and facilitated the resumption of the menstrual cycle in both PCOS with elevated PRL and prolactinoma cases. In the PCOS group, there was a significant decrease in both total testosterone and DHEA-S levels, whereas in the prolactinoma group, only total testosterone levels were significantly reduced. Moreover, in patients with PCOS with elevated PRL, statistically significant improvements in hirsutism scores were also observed with sufficiently long follow-up durations under CAB therapy. Beyond its established role in PRL suppression, CAB demonstrated promising potential in reducing androgen excess, particularly in PCOS with elevated PRL cases, and improving clinical outcomes, without inducing notable changes in FSH or LH levels.

The results of the present study are consistent with the findings of previous pilot studies investigating the use of DAs in PCOS patients. Studies examining the outcomes of dopamine agonist therapy in PCOS patients have been summarized in Table 4, highlighting their distinctive features and significance. In a study conducted by Paoletti et al., involving a total of 29 women (14 with PCOS and 15 in the control group), 16 participants (8 with PCOS and 8 in the control group) received 0.5 mg of CAB weekly for 4 months. The results demonstrated that CAB effectively reduced PRL levels, controlled androgen levels, and improved menstrual cycles in women with oligomenorrhea or amenorrhea. While a decrease in PRL levels was observed as expected, LH pulsatility remained unaffected, indicating that CAB has a limited effect on LH secretion [13]. In another study by Ajossa et al., which offered an alternative for the effects of CAB in PCOS, 30 participants were examined, including 20 women with PCOS and 10 healthy controls. The study reported that CAB normalized androgen levels, improved reproductive health, and restored menstrual cycles in 70% of women with PCOS. The authors also measured the mean pulsatility of the uterine artery with the Doppler flow-assisted transvaginal ultrasonography before and after CAB treatment of patients and controls. They found a significant decrease in uterine perfusion resistance, with a mean pulsatility index value of 3.14 ± 0.6 before and 2.39 ± 0.5 during treatment. Therefore, they suggested that the reduction in PRL achieved with CAB treatment enhanced uterine perfusion, contributing to the restoration of menstrual cycles [18].

**Table 4:** Summary of published studies evaluating the therapeutic effects of dopamine agonists in patients with PCOS

Authors, reference	Year	Study design	Studied population	Number of patients	Number of controls	Intervention groups	Time points	Outcome measure(s)	*Significance*, main result(s)
Paoletti et al. [13]	1996	Randomized-controlled trial	PCOS vs. control	14	15	-CAB group (*n* = 8 [PCOS], *n* = 8 [control]) vs. -Placebo group (*n* = 6 [PCOS], *n* = 7 [control])	At baseline and at the 4th month	• Menstrual cyclicity • Androgen levels • LH pulcatility	***First study evaluating CAB in PCOS*** -Normalized androgen levels and -Improved menstrual cyclicity (in CAB group compared to placebo)
Ajossa et al. [18]	1999	Prospective randomized trial	PCOS vs. control	20	10	-CAB group (*n* = 10 [PCOS]) vs. -Placebo group (*n* = 10 [PCOS], *n* = 10 [control])	At baseline and at the 3rd month	• Pulsatility index of the uterine artery	***First study evaluating uterine artery pulsatility in CAB-treated patients*** -High resistance in the uterine arteries (in PCOS compared to controls) -Increased uterine perfusion (in CAB-treated group compared to placebo)
Ghaneei et al. [14]	2015	Randomized clinical trial	PCOS with elevated prolactin	110	N/A	-MTF + CAB group (*n* = 55) vs. -MTF + placebo group (*n* = 55)	At baseline and at the 4th month	• Menstrual cyclicity • Androgen levels • Prolactin	***First study evaluating MTF plus CAB in PCOS with elevated prolactin*** -Improved menstrual cyclicity (in MTF + CAB group)
Elsersy MAM [19]	2017	Randomized clinical trial	PCOS with elevated prolactin	250	N/A	-MTF + CAB group (*n* = 127) vs. -MTF + placebo group (*n* = 123)	At baseline and at the 3rd month	• Menstrual cyclicity • Androgen levels • Prolactin	***The study with the largest sample group*** -Higher rate of cycle regulation (in MTF + CAB group)
Hamad et al. [24]	2023	Randomized clinical trial	PCOS with elevated prolactin	75	N/A	-MTF group (*n* = 25) vs. -CAB group (n = 25) vs. -MTF + CAB group (*n* = 25)	At baseline and at the 3rd month	• Menstrual cyclicity • Androgen levels • Pulsatility index of the uterine artery • BMI	-Reduced BMI, testosterone, and LH levels (in MTF + CAB group) -Increased FSH levels (in MTF + CAB group) -Improved endometrial blood flow and ovulation (in MTF + CAB group)
Present study	2025	Retrospective cohort study	PCOS with elevated prolactin and microprol-actinoma	66	N/A	-CAB-treated PCOS group (*n* = 36) vs. -CAB-treated microprolactinoma group (*n* = 30)	At baseline and after the median 3 months of CAB treatment	• Menstrual cyclicity • Androgen levels • Hirsutism scores	***The study demonstrating CAB treatment efficacy in a real-world setting*** -Reduced androgen levels, -Improved menstrual cyclicity, -Improved FG scores in CAB-treated PCOS patients with elevated prolactin

*BMI* body mass index, *CAB* cabergoline, *FG* ferriman-gallwey, *FSH* follicule-stimulating hormone, *LH* luteinizing hormone, *MTF* metformin, *PCOS* polycystic ovary syndrome

Previously, randomized controlled trials have been conducted involving treatment protocols that include combinations of CAB and metformin in PCOS patient groups. In a study by Ghaneei et al. involving 110 PCOS women with increased serum PRL concentration, half of the participants received a combination of 1 g/day metformin and 0.5 mg/week CAB for 4 months, while the other half received 1 g/day metformin with a placebo. The CAB+metformin group exhibited significant reductions in PRL, DHEA-S, and total testosterone, whereas the metformin+placebo group showed decreases in only in DHEA-S and total testosterone levels without in PRL. The extent of androgen reduction was comparable between the two groups. However, menstrual irregularities improved more significantly in the CAB+metformin group, with 58.2% achieving menstrual regularity compared to 36.4% in the metformin+placebo group (*p* = 0.02) [14]. Similar findings were reported in another randomized clinical study involving 250 hyperprolactinemic women with PCOS. Of these, 127 received 1 g/day metformin alongside 0.5 mg/week CAB, while 123 were given metformin and a placebo. Both groups showed significant reductions in BMI, total testosterone, and DHEA-S levels following treatment. The change in androgen levels between the two groups were similar. Notably, menstrual irregularities improved significantly more in the combination therapy group compared to the metformin+placebo group (59.8% vs. 29.3%, *p* < 0.001). This study also suggested that the androgen reduction in the CAB group may be directly related to the decrease in PRL levels [19].

Although these studies have demonstrated that CAB can reduce androgen levels in women with PCOS, the underlying mechanism of this effect remains unclear. Ajossa et al. and Paoletti et al. have suggested that the reduction in androgen levels may be linked to PRL inhibition [13, 18]. However, in the current study, while both PRL and DHEA-S levels decreased in the majority of the 30 PCOS patients, four patients exhibited a decrease in PRL without a corresponding drop in DHEA-S levels. Similarly, only one patient showed no reduction in total testosterone despite a decrease in PRL levels. These findings suggest that normalizing hyperprolactinemia alone may not be sufficient to correct hyperandrogenism, indicating that additional mechanisms likely contribute to the regulation of androgen levels.

PCOS remains a disorder with an incompletely understood pathogenesis, with gonadotropin abnormalities being one of the key mechanisms implicated in its development. Several studies in the literature attribute the disrupted LH/FSH ratio in PCOS to mechanisms that result in rapid LH stimulation and elevated LH concentrations [20, 21]. In healthy women, the LH/FSH ratio typically ranges between 1 and 2, whereas in women with PCOS, this ratio is often elevated, reaching as high as 2 or 3 [22]. Prelevic et al. aimed to evaluate the effects of DAs, particularly L-dopa and bromocriptine, on serum PRL, LH, and FSH levels in women with PCOS. They compared three groups: hyperprolactinemic PCOS patients, normoprolactinemic PCOS patients, and healthy controls. Both L-dopa and bromocriptine led to a significant reduction in LH levels in the hyperprolactinemic PCOS group compared to the normoprolactinemic subgroup (*p* < 0.01) and the control group (*p* < 0.05). No significant changes were observed in FSH levels across all groups. Notably, this study did not evaluate androgen levels following DA administration [20]. In a related study, Chapman et al. further explored the effects of bromocriptine on LH pulsatility in PCOS patients. In their study, 10 women with PCOS were treated with 10 mg/day bromocriptine for one year, resulting in a significant decrease in mean serum LH levels from 17.4–11.2 IU/L after 12 months (*p* < 0.03). Additionally, menstrual frequency improved, and significant reductions were observed in testosterone and DHEA-S levels, although androstenedione levels remained unchanged [23].

While earlier studies by Prelevic and Chapman focused on the effects of dopamine agonists on LH levels in PCOS, a more recent study by Hamad et al. examined 75 hyperprolactinemic women with PCOS, divided into three treatment groups: metformin alone (500 mg twice daily), CAB alone (0.5 mg once weekly), and a combination of both treatments for 90 days. In the combination group, LH levels significantly decreased and FSH levels increased, while the reductions in LH levels in the metformin and CAB monotherapy groups were not significant. They suggested that the combination therapy was more effective in lowering LH levels than either treatment alone. Although the study did not provide a direct explanation for the mechanism of LH reduction, the combination group also showed a significant decrease in testosterone levels, unlike the monotherapy groups [24].

Before confirming a diagnosis of hyperprolactinemia in a patient with PCOS, the possibility of a pituitary adenoma must be excluded. This is crucial for predicting the duration and management of any potential DA therapy. Nonetheless, differentiating hyperprolactinemia in PCOS patients can be challenging and often requires additional evaluations. One study suggested that a PRL threshold of 52.9 ng/mL is useful in distinguishing PCOS with elevated PRL from prolactinoma and argued that pituitary MRI may not be necessary for patients with PRL levels below this cut-off who present with a strong clinical suspicion of PCOS [9]. On the other hand, the present study demonstrated that in hyperprolactinemic women with both microprolactinomas and PCOS, CAB therapy was effective in reducing androgen levels. This finding suggests that normalizing PRL levels alone, regardless of the underlying etiology, plays a significant role in restoring menstrual cycles.

The current study has certain limitations that should be acknowledged. First, its retrospective, single-center design may impose some constraints. Second, the small sample size is another important limitation. Third, the administration of CAB therapy specifically to hyperandrogenemic PCOS cases with elevated PRL, in the absence of a clear indication, might have introduced selection bias. Additionally, the short follow-up period prevented a comprehensive assessment of the long-term effects and safety of CAB. In particular, considering that a hair follicle cycle takes 3–5 months to complete, the follow-up duration in the present study is insufficient for a comprehensive evaluation of hirsutism. Nonetheless, the significant improvement observed in 9 PCOS patients with a median follow-up duration of 4.5 months is promising. In interpreting the observed improvement in hirsutism, one must also consider real-life factors such as cosmetic hair removal practices. Although such interventions were not routinely documented, clinicians routinely inquire about them and aim to score hirsutism objectively, minimizing potential bias. However, the retrospective nature of the study limited our ability to fully control for this variable, which is acknowledged as a limitation. On the other hand, this study is the first to report real-world outcomes of CAB therapy in PCOS patients, with the findings evaluated in conjunction with microprolactinoma cases.

In conclusion, CAB is effective in lowering PRL and androgen levels while improving menstrual regularity in both PCOS and prolactinoma patients, highlighting its potential as a valuable therapeutic option for PCOS with elevated PRL cases. The mechanisms by which DAs exert their effects on PCOS remain complex and cannot be attributed to a single pathway.

## Data Availability

No datasets were generated or analysed during the current study.
